# Dietary intake, nutritional adequacy and food sources of vitamins involved in the methionine-methylation cycle from Spanish children aged one to <10 years: results from the EsNuPI study

**DOI:** 10.3389/fnut.2023.1248908

**Published:** 2023-12-14

**Authors:** Teresa Partearroyo, María de Lourdes Samaniego-Vaesken, Paula Rodríguez-Alonso, María José Soto-Méndez, Ángela Hernández-Ruiz, Ángel Gil, Gregorio Varela-Moreiras

**Affiliations:** ^1^Grupo USP-CEU de Excelencia "Nutrición Para la Vida (Nutrition for Life)", Departamento de Ciencias Farmacéuticas y de la Salud, Facultad de Farmacia, Universidad San Pablo-CEU, CEU Universities, Boadilla del Monte, Spain; ^2^Spanish Nutrition Foundation (FEN), Madrid, Spain; ^3^Iberoamerican Nutrition Foundation (FINUT), Granada, Spain; ^4^Biomedical Research Center, Institute of Nutrition and Food Technology "José Mataix", University of Granada, Granada, Spain; ^5^Physiopathology of Obesity and Nutrition (CIBEROBN), Instituto de Salud Carlos III (ISCIII), Madrid, Spain; ^6^Department of Biochemistry and Molecular Biology II, University of Granada, Granada, Spain; ^7^Instituto Biosanitario de Granada (IBS Granada), Granada, Spain

**Keywords:** folates, B_12_, B_6_, pediatrics, Spanish children, dietary habits, nutrition assessment, pediatric nutrition

## Abstract

**Background:**

Methionine-methylation cycle and the derived critical functions during infancy are key regulated by folates, vitamins B_12_, and B_6_. At present in Spain, there is an absence of studies that assess the intakes and dietary sources of total folates and B_12_ by children consuming all types of milks and those regularly consuming adapted milk formulas. Thus, our aim was to evaluate folates intakes alongside with vitamins B_6_ and B_12_ while describing their major dietary contributors in Spanish children aged one to <10 years.

**Methods:**

A total of 1,448 children aged between 1 and 10 years (49.7% girls and 50.3% boys) from the EsNuPI, a prospective cross-sectional study, were allocated into two cohorts: one Spanish Reference Cohort (SRS) of the general population (*n* = 707), and another including children consuming adapted milks called Adapted Milk Consumers Cohort (AMS) (*n* = 741) completed two 24 h dietary recalls used to estimate their nutrient intakes and to compare them to the European Food Safety Authority (EFSA) Population Reference Intakes.

**Results:**

The median intake of vitamin B_6_ was 1.35 (1.06–1.70) mg/day in the SRS and 1.45 (1.17–1.79) mg/day in the AMS, being significantly higher in the AMS for all age-groups. Prevalence of adequacy for vitamin B_6_ in the SRS and AMS was 97.7 and 98.7%, respectively. Total folates intakes in the AMS were significantly higher (*p* ≤ 0.001) in all age groups than in the SRS, independently of age. In addition, the prevalence of adequacy for folates intakes in all groups was more than 60%. Vitamin B_12_ intake increased with age independently of the type of milk consumed. The prevalence of adequacy for vitamin B_12_ was highly compliant by all population groups. The major contributors to vitamin B_6_ were milk and dairy products being significantly higher in AMS than SRS (*p* ≤ 0.001). The highest contributors to folates intakes were milk and dairy products, cereals, vegetables, and fruits in both groups whereas for vitamin B_12_ in the SRS sample were milk and dairy products followed by meat and meats products and for adapted milks, were milk and dairy products, followed by eggs, then meat and meats products.

**Conclusion:**

A satisfactory prevalence of adequacy for vitamins B_6_, and B_12_ amongst the Spanish children population was observed, which was not the case for folates, regardless of the dietary group evaluated. Nevertheless, a possible strategy to increase folate intake among the youngest children is to increase the consumption of milk and dairy products within a healthier dietary pattern, as these may contribute significantly to the vitamin needs of the infant population.

## 1 Introduction

**Figure 1:**
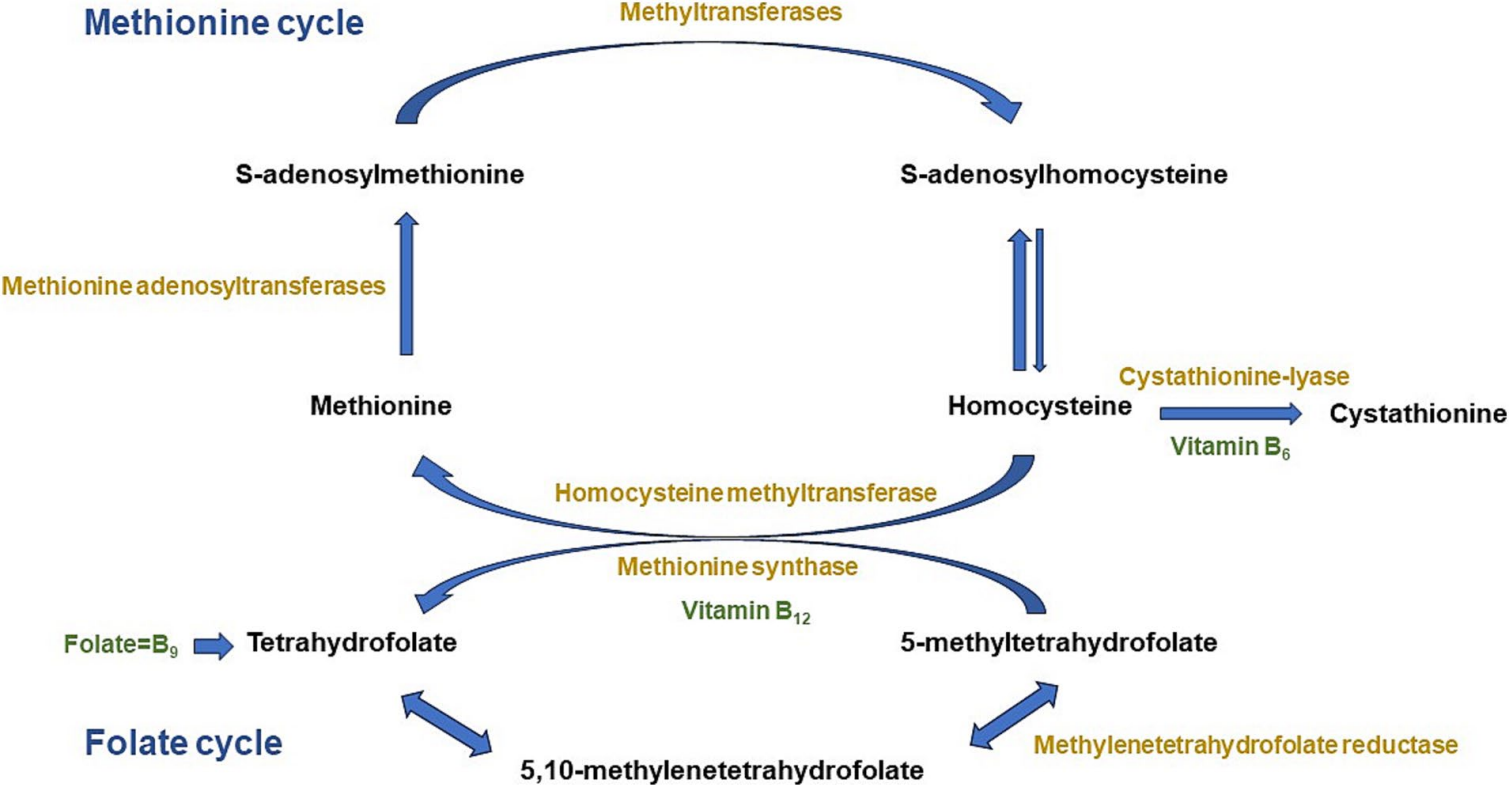
Methionine and folate metabolism and connecting pathways. Adapted from Partearroyo et al. (6).

More than 20 years after the publication of the breakthrough study that revolutionized supplementation policies worldwide, the Medical Research Council Vitamin Study (1), today it is widely recognized that folates deficiency during the periconceptional period can contribute to the development of Neural Tube Defects (NTDs) in the fetus or even affect/impact the newborn with incapacitant conditions such as spina bifida, anencephalia or even heart defects (2, 3). In addition, the availability of the omega-3 long-chain polyunsaturated fatty acid docosahexaenoic, also needed for normal brain development, might be limited by an impaired one-carbon metabolism (4) for which folates, vitamins B_12_ and B_6_ are key nutritional regulators. Vitamin B_6_ and folates are closely working toward maintenance of essential developmental and adult processes (5). In fact, vitamin B_6_ active form, pyridoxal 5′-phosphate, regulate folates levels and it is a necessary cofactor for enzymes involved in the folates metabolism (Figure 1) (6, 7). Therefore, vitamin B_6_, folates, and vitamin B_12_ deficits during child growth might additionally compromise brain development and cognitive function in the future (8). Moreover, the American Academy of Pediatrics has issued a statement advocating a focus on key nutrients for brain development, such as folic acid, vitamin B_6_ and B_12_, among others (9).

Folates is the generic term that encompasses several vitamers with the activity of pteroylglutamic acid or vitamin B_9_ (10), that plays a crucial role in the metabolism of the developing child. Its functions include red blood cell formation and DNA synthesis through the one-carbon cycle (11). Folic acid (FA) is the synthetic form that has a higher bioavailability and which is commonly used in dietetic supplements and fortified food products such as wheat flour (e.g., mandatory fortification) or breakfast cereals (e.g., mainly, voluntary fortification) (12). Like folates, vitamin B_6_ encompasses a neuroprotective role being a cofactor for glutathione and decreasing the brain’s susceptibility to oxidative stress-based damage (13) and its deficiency contributes to neurodegeneration (14). Vitamin B_12_ or cyanocobalamin is also involved in red blood cell formation (11) but while folic acid is effective in the treatment of pernicious and other macrocytic anemias (15), vitamin B_12_ can prevent megaloblastic anemia (16). The interconnection of these vitamins within the one-carbon cycle has been the subject of several studies in the past and still at present, although there is great controversy regarding populations at risk for their deficiency, of which infants are a crucial group to follow up. On the other hand, a strong reason for concern amongst public health authorities about excessive FA intakes has been unmetabolized FA in non-target groups as it was shown that it could accelerate or promote cancer, specifically colorectal (17). Thus, the European Union has not implemented mandatory fortification of any food product and only voluntary fortification takes place. Previous research by Samaniego-Vaesken et al. (18) described the typology and level of fortification available in the Spanish market: most products being ready-to eat breakfast cereals and confectionery products (usually high in added sugars and salt). However, other products such as FA fortified milk and beverages were also frequent. Unfortunately, consumption of these products remains unknown as we lack updated information of fortified food intakes by the Spanish population, including toddlers and children. A study by Egan et al. (19) in Ireland (a voluntary fortification market very similar to the Spanish one in terms of types of products and levels of fortification) showed that voluntary FA fortification levels of food products in Ireland were declining since 2014, and that this might pose implications for FA intakes.

Paradoxically, up to date we did not have a representative sample of the pediatric population like the one provided by the EsNuPi study [Estudio Nutricional en Población Infantil Española (Nutritional Study in Spanish Pediatric Population)] performed in Spanish children aged one to <10 years old (20) in order to update and improve the knowledge on micronutrient intakes related to the methionine cycle.

The novelty of the present work relies on data of micronutrients related to the methionine cycle that has not been previously published elsewhere and does not contain excessive overlap with previously published data from the ESNUPI study. Intakes of these vitamins have not been published in a sample of this age range (one to <10 years old) which is representative of the Spanish population.

Therefore, there is an urgent need for a better and more updated knowledge of the intakes of vitamins related to the methionine cycle in the Spanish pediatric population in order to prevent and/or delay the adverse effects derived from inadequate intakes in this stage of life. In fact, in the present study our objective was to assess folates intakes alongside vitamins B_6_ and B_12_ and to describe their major dietary contributors as well as to study the differences between infants who usually consume adapted milks and those consuming all types of milk.

## 2 Materials and methods

### 2.1 Study design and sample

The EsNuPI study is a prospective, cross-sectional, observational study, that took place between October 2018 and January 2019. Full design, protocol and methodology have been published elsewhere (20). The EsNuPI study evaluated the dietary patterns and nutrient intake, in addition to physical activity and sedentary behaviors of Spanish children that lived in urban areas with >50,000 inhabitants, from nine geographical areas as established by Nielsen. These urban areas represent 1.8% of the total number of municipalities of Spain and 52.6% of the total Spanish children population of one to <10 years old (21). We compared two subsamples aged one to <10 years old, one comprising the urban non-vegan individuals who consumed all types of milk in the last 12 months, the “Spanish Reference Sample, SRS,” and another called “Adapted Milk Consumers Sample, AMS” of non-vegan subjects from urban areas who only consumed adapted milks in the last 12 months. The “adapted milks” food group included infant formulas, follow-on milk formulas, toddler’s milk formulas (also known as “young children milk formulas” and in Spain called “growing up” milk formulas) and fortified and enriched milk formulas [i.e., with added docosahexaenoic acid (DHA), calcium, vitamin D, and/or iron]. Originally, the estimated sample consisted of 1,500 individuals, and the sample errors were ± 2.52% and ± 2.59%, respectively, for a 95.5% confidence level and estimation of equally probable categories (*p* = *q* = 50%), considering a universe of 2,205,646 children. Final recruitment included a total of 1,514 children (*n* = 742 SRS; *n* = 772 AMS) and 1,448 individuals finished the study (95.6% response rate). The EsNuPI study was performed under the declaration of Helsinki, approved by the University of Granada ethical committee (No. 659/CEIH/2018), and registered in ClinicalTrials.gov (Unique Protocol ID: FF01/2019).

### 2.2 Data collection

A face-to-face interview was completed by participants who provided sociodemographic information and a first 24-h dietary recall (24-h DR). Then, after a week, a second 24-h DR was answered by participants by telephone. The two 24- DR included both, a holiday and school day. MADISON MK (TELECYL. S.A.) collected all personal data of respondents in order to manage and carry out the survey. MADISON MK is responsible and adapted to the new General Data Protection Regulation of the European Union (EU) 2016/679. Each participant provided express consent to participate in the survey. Once the data collection and the survey verification work were finished, the files obtained were anonymized for their processing. Fieldwork was undertaken from October 2018 to January 2019. Further information has been provided by (20).

#### 2.2.1 Data collection instruments

A general questionnaire was used to collect the following variables in the first interview: place and date of birth, gender, academic level of parents or caregivers (elementary or less/secondary/university/higher education), place of residence, family income level, lifestyle, activity patterns, and sedentary behaviors. Height and weight data were declared by parents or caregivers, based on the child’s pediatric health card.

### 2.3 Procedures, dietary survey and data collection

Two independent 24-h DR to determine children’s dietary intake (proxy report) were provided by parents or caregivers; that included a week and a weekend day (non-consecutive), one being face-to-face and the other by telephone a week later.

Dietary intake was thoroughly described by including the ingredients and methods of preparation that were organized as mealtimes (breakfast, mid-morning, lunch, midafternoon, dinner, and other moments) in order to calculate energy and nutrient distribution during the day.

Assessment of children intakes encompasses many methodological difficulties such as misreporting. Its calculation was performed by the protocol proposed by the European Food Safety Authority (EFSA), based on the work by Goldberg (22) and Black (23) that evaluates the reported energy intake (EIrep) against the energy requirements. In the present manuscript, results included were not adjusted for misreporting, as in the previous study by from Madrigal et al. (24) exclusion of misreporters in the EsNuPI population showed no significant differences in the total energy intake (TEI) and the distributions of relative macronutrient intakes. Thus, it was assumed that it does not significantly modify the results and conclusions of this study.

The adequate completion of the survey was facilitated by resources such as the “Tables of common home measures and habitual portion sizes for the Spanish population” (25) and the “Photo guide of common portions sizes of Spanish foods” (26) that were used by interviewers. In order to calculate the food, beverage, energy and nutrient intakes, the “VD-FEN 2.1” software, a Dietary Evaluation Program developed by the Spanish Nutrition Foundation (FEN) was used. Total folates, B_6_, and B_12_ intakes were obtained by calculating the prevalence of adequacy for these micronutrients which was defined as the percentage of the studied population (%) that showed intakes of these vitamins above 80% of the Population Reference Intakes (PRIs) established by the EFSA (27).

### 2.4 Statistical analysis

The collection of reported dietary intake data resulted in a total of 746 food items that were categorized into 18 food groups as described (20) and then converted into energy and nutrient data for analysis. All estimates were made from an average across the 2 days of diet recording. The normality of the distribution of the variables was obtained with the Kolmogorov-Smirnoff normality test. Median and interquartile range (IQR) were used for continuous variables and frequencies and percentages for categorical variables, to describe folates, vitamins B_6_, and B_12_ intake by type of sample (SRS and AMS) gender and age groups. The Mann–Whitney *U*-test was used for comparisons between SRS and AMS by gender and age group. Kruskal–Wallis Test and the Dunn Test were used to adjust for multiple comparisons and adjustment of the value of *p* with Bonferroni correction were used to calculate differences among each age group within samples. The level of significance was established at *p* < 0.05. IBM SPSS 27.0 (IBM Corp., Armonk, NY, USA) was used to perform data analysis.

## 3 Results

A total of 1,448 children between the ages of 1 to <10 years (49.7% girls and 50.3% boys) completed the study. Their anthropometric, sociodemographic, and physical activity characteristics have been previously published (24, 28).

### 3.1 Folates intakes and prevalence of adequacy in children

Total folates intakes, as well as the prevalence of adequacy are shown in Table 1 for each cohort, by gender and age. When results were analyzed by cohorts, we found that total folates intakes in the AMS were significantly higher (*p* ≤ 0.001) in all age groups than in the SRS, independently of age. In addition, regarding the prevalence of adequacy for folates intakes (% population above 80% PRI), we found that more than 60% of individuals complied with the recommendations within all groups. However, it is important to note that the prevalence of adequacy to recommended folates intakes for this Spanish young population was significantly higher in the adapted milk cohort than in the reference cohort (*p* ≤ 0.001), except for the 1 to <3 years group, which did not differ statistically between cohorts.

**Table 1:** Folates intakes and prevalence of adequacy (percentage of population > 80% PRI) by gender and age group in the Spanish Pediatric Population (EsNuPI) study.

Group	Group by age	Total population			Boys			Girls		
		*n*	Folates (μg/d)	% >80 PRI EFSA	*n*	Folates (μg/d)	% >80 PRI EFSA	*n*	Folates (μg/d)	% >80 PRI EFSA
SRS	1 to <3 years	162	150.9^a^ (106.8–199.7)	95.1^a^	84	147.5^a^ (108.1–188.9)	92.9^a^	78	151.8 (105.8–210.3)	97.4^a^
	3 to <6 years	244	160.0^a,b^ (126.5–219.6)	72.1^b^	122	165.8^a,b^ (126.9–221.2)	76.2^a,b^	122	156.2 (125.0–215.3)	68.0^b^
	6 to <10 years	301	177.7^b^ (135.0–229.5)	61.8^c^	151	180.9^b^ (133.7–234.7)	64.9^b^	150	173.6 (135.2–223.3)	58.7^b^
AMS	1 to <3 years	294	177.3***^,a^ (137.2–222.1)	96.9^a^	144	176.6***^,a^ (135.0–222.2)	95.8	150	178.1**^,a^ (138.9–222.6)	98.0^a^
	3 to <6 years	262	230.5***^,b^ (171.8–310.6)	93.1***^,a,b^	128	232.3 ***^,b^ (171.8–312.6)	94.5***	134	229.6 ***^,b^ (171.3–307.2)	91.8***^,a,b^
	6 to <10 years	185	279.7***^,c^ (209.7–393.4)	87.6***^,b^	99	289.3 ***^,c^ (217.4–403.7)	88.9***	86	275.8 ***^,c^ (204.2–352.0)	86.0***^,b^

Population Reference Intakes (PRIs) (27) Spanish Reference Cohort (SRS) and Adapted Milk Consumers Cohort (AMS). Values are presented as median (interquartile range) and percentage. Values that do not share superscript are significantly different between age groups for each gender and in each cohort type (*p* ≤ 0.05; Kruskal–Wallis test and the Dunn test to adjust for multiple comparisons and adjust the value of p with Bonferroni correction) and asterisk indicate a statistically significant difference between cohort type difference vs reference cohort (**p ≤ 0.01; ***p ≤ 0.001; Mann–Whitney’s *U* test).

Conversely, we observed that when results were analyzed by age groups, as expected, total folates intakes were significantly higher amongst older groups independently of the type of milk consumed, except in the SRS, which had a similar total folates intake regardless of gender and age-group to which they belonged.

When evaluating the proportion of children who reached more than 80% of the PRI for folates by age and gender, we found that, when segmented by gender, in all groups >55% of the individuals met the recommendations. In addition, it is worth emphasizing that the children who consumed adapted milk showed a higher compliance with the recommendations than those from the reference group, except those from early ages (1 to <3 years), who had a high level of compliance with recommendations independently the type of milk they consumed.

### 3.2 Vitamin B_6_ intakes and prevalence of adequacy in children

Table 2 presents the daily intake of vitamin B_6_ in the study population and separately by age and gender groups, as well as differentiating between the SRS and the AMS. The median intake of vitamin B_6_ was 1.35 (1.06–1.70) mg/day in the SRS and 1.45 (1.17–1.79) mg/day in the AMS cohort. Vitamin B_6_ intakes were significantly higher in the AMS for all age groups with respect to SRS. However, no significant differences were observed regarding vitamin B_6_ intakes within age groups across the whole sample. Prevalence of adequacy for vitamin B_6_ in the SRS and AMS was 97.7 and 98.7% respectively, in line with the EFSA criteria (Table 2). Furthermore, the prevalence of adequacy for vitamin B_6_ estimated by the EFSA criteria was significantly higher in AMS, independently of age (Table 2).

**Table 2:** Vitamin B_6_ intakes and prevalence of adequacy (percentage of population > 80% PRI) by gender and age group in the Spanish Pediatric Population (EsNuPI) study.

Group	Group by age	Total population			Boys			Girls		
		*n*	Vitamin B_6_ (μg/d)	% >80 PRI EFSA	*n*	Vitamin B_6_ (μg/d)	% >80 PRI EFSA	*n*	Vitamin B_6_ (μg/d)	% >80 PRI EFSA
SRS	1 to <3 years	162	1.23 (0.94–1.54)	96.9	84	1.14 (0.92–1.50)	95.2	78	1.30 (0.96–1.61)	98.7
	3 to <6 years	244	1.37 (1.01–1.70)	98.4	122	1.40 (1.09–1.67)	99.2	122	1.34 (1.03–1.78)	95.7
	6 to <10 years	301	1.41 (1.13–1.77)	97.7	151	1.42 (1.11–1.86)	98.0	150	1.39 (1.13–1.72)	97.3
AMS	1 to <3 years	294	1.36** (1.08–1.60)	99.7*^,a^	144	1.38** (1.10–1.60)	99.3*	150	1.31 (1.05–1.62)	100.0^a^
	3 to <6 years	262	1.54*** (1.26–1.92)	99.2^a^	128	1.51** (1.28–1.9)	100.0	134	1.58*** (1.24–1.95)	98.5^a,b^
	6 to <10 years	185	1.56* (1.25–1.92)	96.2^b^	99	1.59 (1.25–1.96)	97.0	86	1.48 (1.23–1.86)	95.3^b^

Population Reference Intake (PRI) (27) Spanish Reference Cohort (SRS) and Adapted Milk Consumers Cohort (AMS). Values are presented as median (interquartile range) and percentage. Values that do not share superscript are significantly different between age groups for each gender and in each cohort type (*p* ≤ 0.05; Kruskal–Wallis test and the Dunn test to adjust for multiple comparisons and adjust the value of *p* with Bonferroni correction) and asterisk indicate a statistically significant difference between cohort type difference vs reference cohort (*p ≤ 0.05; **p ≤ 0.01; ***p ≤ 0.001; Mann–Whitney’s *U* test).

### 3.3 Vitamin B_12_ intakes and prevalence of adequacy in children

Table 3 shows the obtained daily intake levels of vitamin B_12_ by age group, which increased with age independently of the type of milk consumed. When comparing age subgroups, we detected important differences in B_12_ vitamin intakes amongst boys aged 1 to <3 years, where the AMS had lower vitamin B_12_ consumption (*p* ≤ 0.05). Furthermore, the potential prevalence of adequacy for B_12_ was above 80% PRI in the total study population according to the European PRI (Table 3). Also shown in Table 3 are the results by the total population and age group. When data were analyzed stratified by age group, we found that the prevalence of adequacy for vitamin B_12_ in accordance with the European PRI was highly compliant by all population groups (>95% compliance) but was significantly higher in the AMS children aged 1 to 3 years vs. the SRS group.

**Table 3:** Vitamin B_12_ intakes and prevalence of adequacy (percentage of population above 80% PRI) by gender and age group in the Spanish Pediatric Population (EsNuPI) study.

Group	Group by age	Total population			Boys			Girls		
		*n*	Vitamin B_12_ (μg/d)	% >80 PRI EFSA	*n*	Vitamin B_12_ (μg/d)	% >80 PRI EFSA	*n*	Vitamin B_12_ (μg/d)	% >80 PRI EFSA
SRS	1 to <3 years	162	2.6 (1.9–3.6)	96.9	84	2.7 (1.9–3.7)	95.2	78	2.6 (1.9–3.2)	98.7
	3 to <6 years	244	3.2 (2.2–4.3)	98.8	122	3.3 (2.3–4.4)	99.2	122	3.1 (2.2–4.0)	98.4
	6 to <10 years	301	3.5 (2.6–4.6)	99.9	151	3.7 (2.5–4.8)	99.3	150	3.4 (2.6–4.4)	98.7
AMS	1 to <3 years	294	2.3 (1.8–3.2)	99.7*	144	2.4* (1.9–3.2)	99.3*	150	2.3 (1.7–3.3)	100.0
	3 to <6 years	262	3.0 (2.3–3.9)	99.2	128	3.0 (2.3–3.7)	99.2	134	3.2 (2.3–4.0)	99.3
	6 to <10 years	185	3.3 (2.4–4.6)	98.4	99	3.3 (2.4–4.6)	98.0	86	3.2 (2.3–4.6)	98.8

Population Reference Intakes (PRIs) (27) Spanish Reference Cohort (SRS) and Adapted Milk Consumers Cohort (AMS). Values are presented as median (interquartile range) and percentage. Values that do not share superscript are significantly different between age groups for each gender and in each cohort type (*p* ≤ 0.05; Kruskal–Wallis test and the Dunn test to adjust for multiple comparisons and adjust the value of *p* with Bonferroni correction) and asterisk indicate a statistically significant difference between cohort type difference vs reference cohort (*p ≤ 0.05; Mann–Whitney’s *U* test).

### 3.4 Contribution of food and beverage groups to folates intakes

**Figure 2:**
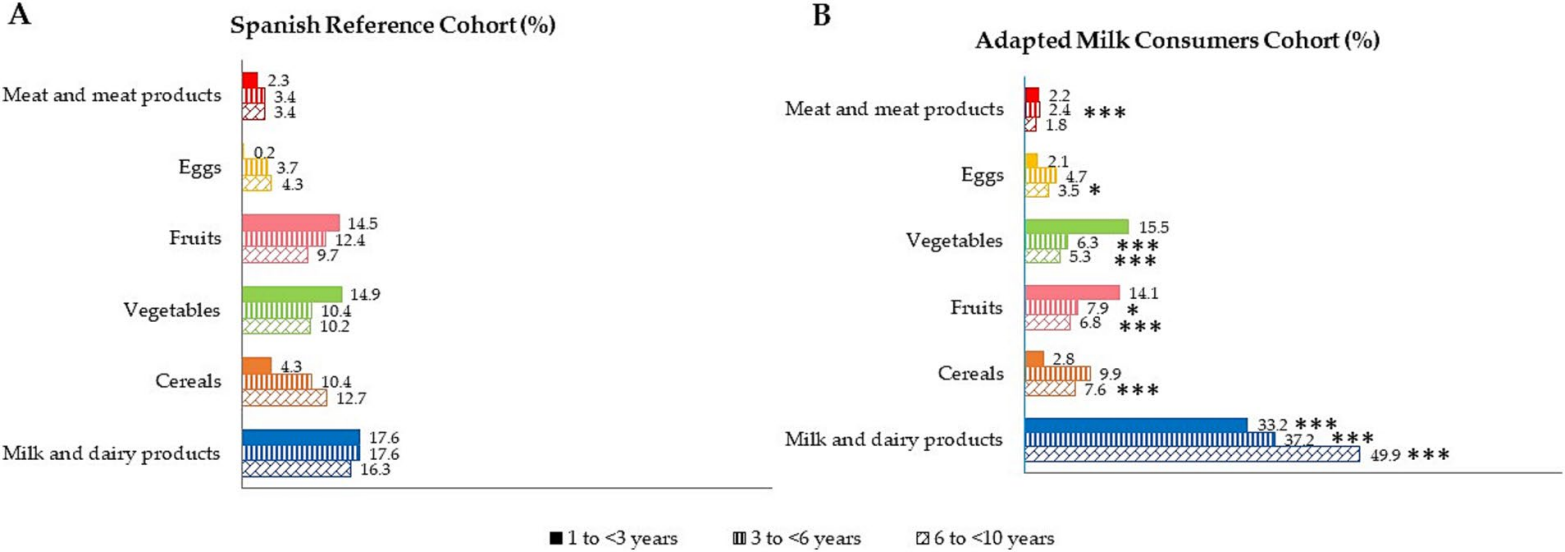
Dietary food and beverage groups contributing to total folates intakes (%) from the EsNuPI study population (“Spanish Pediatric Population”) in both Spanish Reference Cohort **(A)** and the Adapted Milk Consumers Cohort **(B)**. *p ≤ 0.05 compared to the reference cohort (Mann-Wllitney test). ***p≤0.001 compared to the reference cohort (Mann-Wllitney test). Only foods contributing to folates intakes of the population have been included.

Figure 2 shows and categorizes the main sources of folates intakes by age group for both samples, amongst children from the EsNuPI study. Data are presented as the median of proportional contribution and only for those foods which contributed at least 1% to folates intakes of the population.

The highest contributors to folates intakes were milk and dairy products, cereals, vegetables, and fruits, in the whole pediatric population. For children in the AMS aged 1 to 3 years, a significantly different contribution was observed, with a higher contribution to folates from the milk and dairy products group AMS compared to the SRS (*p* ≤ 0.001). Equally, AMS children aged 3 to 6 years had a higher contribution of folates from milk and dairy products (*p* ≤ 0.001), and a lower folates contribution from vegetables (*p* ≤ 0.001), meat and meats products (*p* ≤ 0.001), and fruits (*p* ≤ 0.05) than SRS children of the same age. The older age group of the AMS presented a higher contribution of folates from milk and dairy products (*p* ≤ 0.001) and lower contribution from cereals (*p* ≤ 0.001), meat and meats products (*p* ≤ 0.001), fruits (*p* ≤ 0.001), eggs (*p* ≤ 0.05), and vegetables (*p* ≤ 0.001) than their counterparts of the SRS.

### 3.5 Contribution of food and beverage groups to vitamin B_6_ intakes

**Figure 3:**
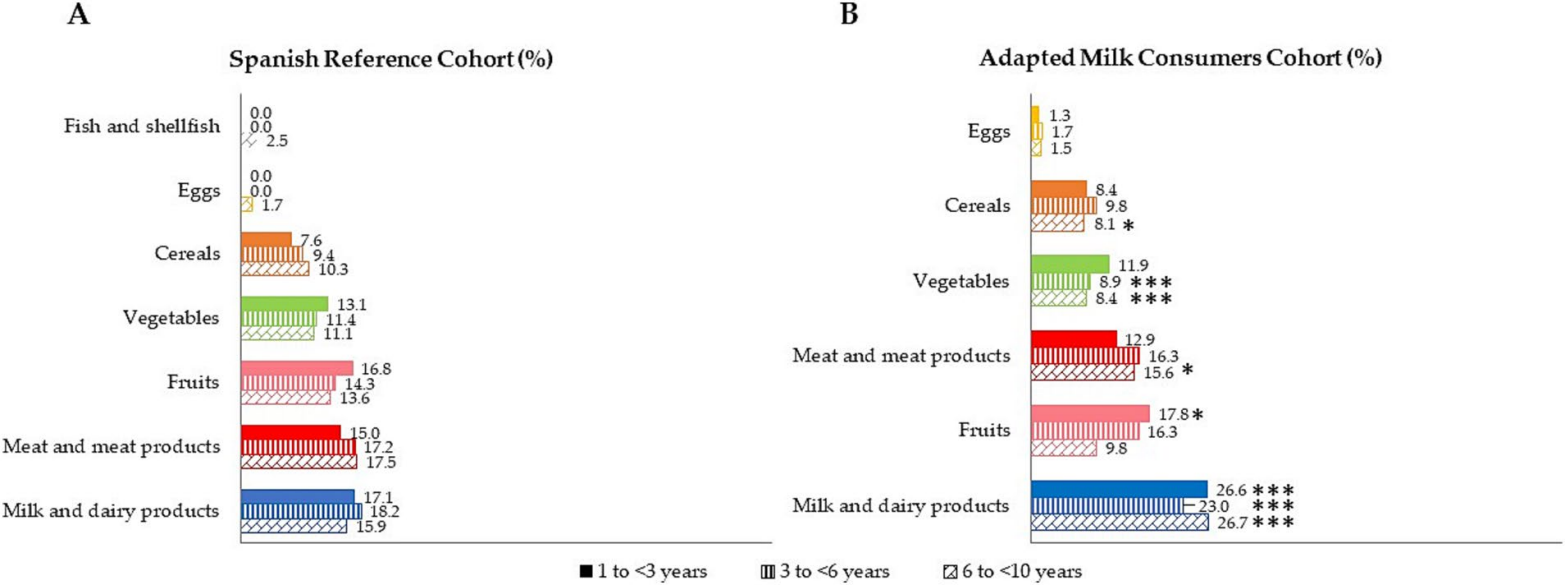
Dietary food and beverage groups contributing to total vitamin B_6_ intakes (%) from the EsNuPI study population (“Spanish Pediatric Population”) in both Spanish Reference Cohort **(A)** and the Adapted Milk Consumers Cohort **(B)**. *p≤0.05 compared to the reference cohort (Mann-Wllitney test). ***p≤0.001 compared to the reference cohort (Mann-Wllitney test). Only foods contributing to vitamin B_6_ intakes of the population have been included.

The contribution (%) of food and beverage categories to the daily vitamin B_6_ intake is shown, categorized by age group for both samples, in Figure 3. It should be noted that only those foods that contributed at least 1% to the vitamin B_6_ intake of the population have been included. Milk and dairy products were the main contributors to vitamin B_6_ for the whole study sample, being significantly higher in AMS than SRS (*p* ≤ 0.001). The following largest contributors were fruits and meat and meat products, as well as vegetables, with differences between the two cohorts.

### 3.6 Contribution of food and beverage groups to vitamin B_12_ intakes

**Figure 4:**
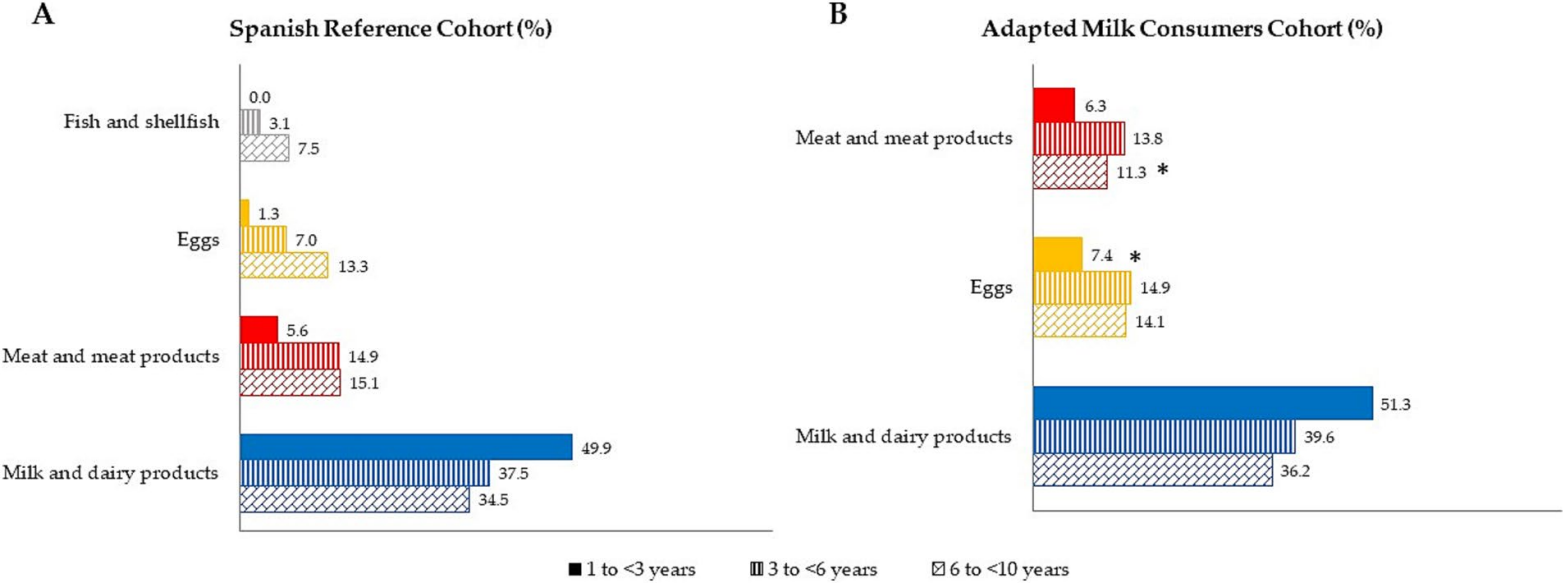
Dietary food and beverage groups contributing to vitamin B_12_ intakes (%) from the EsNuPI study population (“Spanish Pediatric Population”) in both reference cohort **(A)** and the Adapted Milk Consumers Cohon **(B)**. *p≤0.05 compared to reference cohort (Mann-Wllitney test). Only foods contributing >l% to vitamin B_12_ intakes of the population have been included.

The median contribution (%) of food groups to vitamin B_12_ intake, categorized by age group for both samples is shown in Figure 4. Data are presented as the median of proportional contribution and only for those foods which contributed at least 1% to vitamin B_12_ intakes of the population. In particular, food groups with the highest median proportional contribution to total vitamin B_12_ intake in the reference sample (SRS) were firstly milk and dairy products (38.3%), followed by meat and meats products (13.1%). Next, eggs accounted for 8.2% of total vitamin B_12_ intake and, finally, fish and shellfish contributed 4.7%. Moreover, we observed that amongst the sample of adapted milk consumers (AMS), the largest contributors to vitamin B_12_ intakes were milk and dairy products (42.4%), eggs (12.9%), and meat and meat products (11.1%).

When comparing samples by age group (Figure 4), AMS children between 3 and < 6 years obtained higher levels of vitamin B_12_ intake from eggs (*p* ≤ 0.05) than SRS children of the same age. In addition, SRS children from 6 to <10 years had a lower contribution to vitamin B_12_ intake from the meat and meats products group than their counterparts of the AMS (*p* ≤ 0.05).

## 4 Discussion

It is acknowledged that poor nutritional status, especially suboptimal status of folates, and vitamins B_6_ and B_12_ can affect brain function and cognitive performance (29), namely by perturbing one-carbon metabolism, and this can take place in early as well as in later life (30). Children of school age need adequate cognition to obtain higher academic performance (31). As a matter of fact, cognitive processes are substantially related with brain function, physiology and structure (32) and, therefore, its development can be highly dependent on nutrition, physical activity, and social and economic status. For this reason, we studied folates together with vitamins B_6_ and B_12_, to find their intakes and major dietary contributors and also to describe the differences between infants at school age who usually consume adapted milks and those consuming all types of milk. In fact, at the light of the results from the present study, we observed a suitable intake of vitamins B_6_ and B_12_ but not for folates amongst Spanish children. In turn, we observed that children consuming adapted milk products had higher folates compliance.

Back in 2008, comprehensive reviews published by Black (8) and Molloy (33) examined the mechanisms linking folates and vitamin B_12_ deficiency to abnormal behaviors and development in infants (disruptions to myelination and inflammatory processes), as well as the effects resulting from these deficiencies. Prevention of folates and vitamin B_12_ deficiencies is pivotal to this age group and should mostly rely on dietary sources as supplementation is only left to specific situations such as strict vegetarian or vegan children who should supplement B_12_ (34). Vitamin B_12_ sources are almost exclusively obtained from the animal kingdom and, therefore, these groups might develop deficiencies if not adequately supplemented. Indeed, a child who is deficient in vitamin B_12_, will continue to suffer from deficiency symptoms if it is not diagnosed early in infancy. Therefore, school-aged children with B_12_ deficiency could be affected by altered motor development, cognitive disorders, and speech and language skills impairment (35). Folates which play a key role in the metabolism of the developing child (11) and vitamin B_12_ deficiency could result in irreversible damage, such as growth stunting (36). In fact, there is also a growing interest in comparing breast and formula feeding in early infancy and their impact later in life, particularly concerning developing diseases such as obesity, hypertension, hyperlipidemia, and diabetes mellitus (37). It is important to highlight that breast milk and/or infant formula are the only sources of nutrition during early infancy and should provide suitable quantities of energy, water, and all essential nutrients (38).

The magnitude of the folate inadequacy in this study in the SRS group was 27%. It was higher than in the study conducted by Serra-Majem et al. (39), in which when assessing the percentage of population predicted to have inadequate intakes of folates using a probability approach, boys 2–5 years had 2.5% and those 6–9 years had 1.7%; however, girls 2–5 years showed 10.1% and those 6–9 years just 2.1%, and the National Dietary Survey on the Child and Adolescent Population in Spain (ENALIA) (40) their estimates showed that in general, water-soluble vitamin intakes were adequate but in the case of folate >50% of the participants older than 9 years had intakes below the specific sex-age estimated average requirement (EAR). Conversely, the degree of folate inadequacy in the children consuming adapted milk had a higher level of compliance with folates recommendations (93.3%) than those in the reference group, except from early ages (1 to <3 years), who had a high level of compliance with recommendations regardless of the type of milk consumed. Nevertheless, it is important to note that dietary folates intakes amongst infants have been previously assessed in Spain, but not specifically targeting the groups studied within the EsNuPI Study (1 to <10 years), and therefore comparison remains limited (39, 41). In addition, the difference could be related to the difference in the use of different methodologies. Nevertheless, data on dietary folate intake in school-age children at a European level show that there are large proportions of this population that do not meet folate recommendations. Indeed, it is striking that a recent review of nutrient intakes from national dietary surveys of 21 surveys in 18 countries in three regions: two of five Northern European countries (Denmark and Norway); 10 out of 17 Western European countries (Germany, Austria, Belgium, Spain, France, Ireland, Italy, the Netherlands, Portugal and the United Kingdom) and six out of 31 Central and Eastern European countries (Bulgaria, Cyprus, Slovenia, Estonia, Latvia and Turkey), the only age group over 3 years with an adequate total folate intake were Irish children aged 13–14 (42). It has earlier been found that an increase of 10% in folate intake from food could result in a 6% increase in serum folate (43). Therefore, nutritional policy-making bodies should consider the need for fortification of foods with folic acid (FA) targeted to this age segment, review the RDIs and formulate specific recommendations to ensure that school-aged children have adequate intake of this micronutrient. However, these strategies will require careful monitoring to ensure effectiveness. Since, Lewis et al. (44) reported that after fortification in the USA, 26% of this population exceeded the tolerable upper limit. Likewise, Pfeiffer et al. (45) estimated that 43% of children up to 5 years of age had an elevated serum folate concentration. Furthermore, in Spain, we have shown that excesses are a common practice in the fortification of FA in breakfast cereals and dairy products, since total folate values were clearly higher than those declared by manufacturers in most cases (18, 46).

This study found that the prevalence of adequacy for vitamin B_12_, among school-aged children in Spain, based on the European PRI was highly compliant by all population groups (>95% compliance), being only significantly higher in the adapted milk group (AMS) in children aged 1 to 3 years vs. the SRS group, which is consistent with findings from other national or international dietary surveys of children where vitamin B_12_ intakes have also met the recommended nutrient intakes (RNI) for vitamin B_12_ at school age (39, 41, 42). The high vitamin B_12_ adequacy noted in Spanish children may be explained by the dietary patterns followed by the Spanish population, which nowadays includes a high intake of animal products, increasingly distant from the traditional Mediterranean diet (47, 48).

Furthermore, a high proportion of this population group have adequate intakes of vitamin B_6_. This is consistent with previous Spanish study, which have showed mean intakes of 1.4 and 1.6 mg/day for vitamin B_6_ in males (2–5 years and 6–9 years, respectively) and female had intake for this vitamin of 1.3 and 1.5 mg/day (2–5 years and 6–9 years, respectively) (39). More recently, in the ANIBES study (41) the youngest age-groups evaluated were those 9–12 years, so only a limited comparison could be undertaken. In addition, 89.7% of children in the ANIBES met the European recommendations for vitamin B_6_ (49).

Comprehensive information on food sources is key for understanding the strengths/weaknesses as well as the quality of the diet of children from the Spanish population. The major contributors of vitamin B_6_ for children from our study were milk and dairy products, followed by fruits and meat and meat products. Also, it is noteworthy that the contribution of folates from milk and dairy products in the AMS was significantly higher than in the SRS. Indeed, although milk and dairy products are the main sources of macro-and micronutrients and contribute to the general quality of the diet in both children and adults (50–52), it appears that the infant formulas, follow-on milk formulas, or toddler’s milk formulas consumption may contribute more effectively to achieving the PRIs in the infant population. In addition, in the present study the AMS also showed milk and dairy products as the major B_12_ contributors but conversely, egg products were the second group as compared to the SRS were meat and meat products. In fact, it is worth remembering that restrictive diets such as vegetarian or vegan encompass a high risk of inadequate intake owing to the exclusion and/or limitation of foods of animal origin. Even more importantly in this stage of exponential growth and development, vitamin B_12_ deficiency amongst these key population groups has to be taken under consideration by public health authorities (53). It has been estimated that in Europe, the prevalence of vegetarian diets ranges from 1.2 to 1.5% of the population in Portugal and Spain, to about 7% in the United Kingdom and 10% in Germany, although the proportion of vegans is substantially lower (1–3%) all over Europe (54). No specific data are available for infants and children, nonetheless, it is acknowledged that vitamin B_12_ status can be compromised if supplementation is not provided for these children under a strictly vegans diets.

Our data can be compared with those acquired in other European studies in which the proportion of vitamin B_12_ and folates obtained from milk and dairy products was significant. For example, milk and dairy products were the major contributors to Greek school children aged 9–13 years (55). In the multi-ethnic population of the Head Start Mothers study (USA), high milk/low sweetened beverage intakes were associated with higher mean intakes of numerous vitamins and minerals, including folates and B_6_ (56). In Australian preschool children, one-carbon metabolism nutrients (methionine, folates, vitamins B_2_, B_6_, and B_12_) intake was also found highest from dairy products (57).

It is worth underlining and something worrisome that according to our results major folate sources, like vegetables and green-leaf as well as legumes are scarce. Indeed, for all age groups, milk and dairy products was the main folate contributor. Noteworthy the SRS had higher vegetable consumption that the AMS, specially at younger ages. However, in both groups fruit intake was similar.

### 4.1 Strengths and limitations

Amongst the strengths of the EsNuPI study is the novelty of the analysis of a subpopulation representative of the Spanish children aged 1 to <10 years and likewise those who consume adapted milk formulas. The EsNuPI study involves a representative cohort of the Spanish children and an updated source of information on their dietary intakes and patterns and socioeconomic factors. In addition, the 24-h DR information was compiled following the methodology suggested by EFSA (The PAN CAKE-Pilot study) (58).

The study presents, however, several limitations: (1) potential errors in the reported information might have influenced the results of the study questionnaires; (2) children living in urban areas were studied; though, currently, 52.6% of the total Spanish population from 1 to <10 years old live in urban areas; (3) due to the study’s non-invasive design, we did not perform any vitamin analysis on plasma; and (4) this work queried about child’s supplement use [only 2.9% reported the consumption of any type of supplement, compared to the 7–25% reported for Spanish children (40)]. Supplements reported in the 24 h DR were included as part of the food group “cereal-based baby food and supplements.” A specific limitation of studying young children when compared to older ones is that they have limitations in remembering, estimating and cooperating in dietary assessment procedures, this is why caregivers became the main information providers (59). However, one has to consider the bias this might represent for example in school meals or the under or overreporting derived by parents in response to their own dietary beliefs. In fact, there is a tendency amongst parents to overestimate those foods considered healthy and underestimate the least healthy ones. However, as already stated, Madrigal et al. (24) performed an evaluation of misreporting for the EsNuPI study which showed that the exclusion of under or over reporters has no significant influence in children’s TEI, therefore they were included in the present study.

## 5 Conclusion

The results showed that higher compliance with folates recommendations was achieved by children consuming adapted milk products. Finally, across all population groups there was a high compliance with B_12_ and vitamin B_6_ PRI (>95%) based on the European recommendations, being significantly higher in the adapted milk group (AMS) in children aged 1–3 years vs. the SRS group for vitamin B_12_. Likewise, the prevalence of adequacy for vitamin B_6_ estimated by the EFSA criteria was significantly higher in AMS, independently of age. In conclusion, children would largely benefit from healthier dietary patterns increasing the consumption of milk and dairy products, since these may contribute significantly to these vitamin requirements of the infant population. Nevertheless, there is a great need to improve young children’s diet by including high folate sources that contribute to an adequate intake of this vitamin.

## Data availability statement

The raw data supporting the conclusions of this article will be made available by the authors, without undue reservation.

## Ethics statement

The studies involving humans were approved by University of Granada Ethical Committee (No. 659/CEIH/2018), and registered in ClinicalTrials.gov (Unique Protocol ID: FF01/2019). The studies were conducted in accordance with the local legislation and institutional requirements. Written informed consent for participation in this study was provided by the participants’ legal guardians/next of kin.

## Author contributions

GV-M and ÁG: funding acquisition, project administration, and supervision. PR-A, MS-M, ÁH-R, GV-M, and ÁG: investigation. MS-V, TP, ÁG, and GV-M: methodology. TP and MS-V: writing—original draft. TP, MS-V, PR-A, MS-M, ÁH-R, ÁG, and GV-M: writing—review and editing. All authors have read and agreed to the published version of the manuscript.
